# Supplementary figures and images for: Cytokines and appetite-regulating hormones in human milk and associations with infant growth across four sites in a longitudinal cohort: The Mothers, Infants and Lactation Quality Study
Source: PLoS One. 2025 May 8;20(5):e0323204. doi: 10.1371/journal.pone.0323204 (PMC12061169; doi:10.1371/journal.pone.0323204)

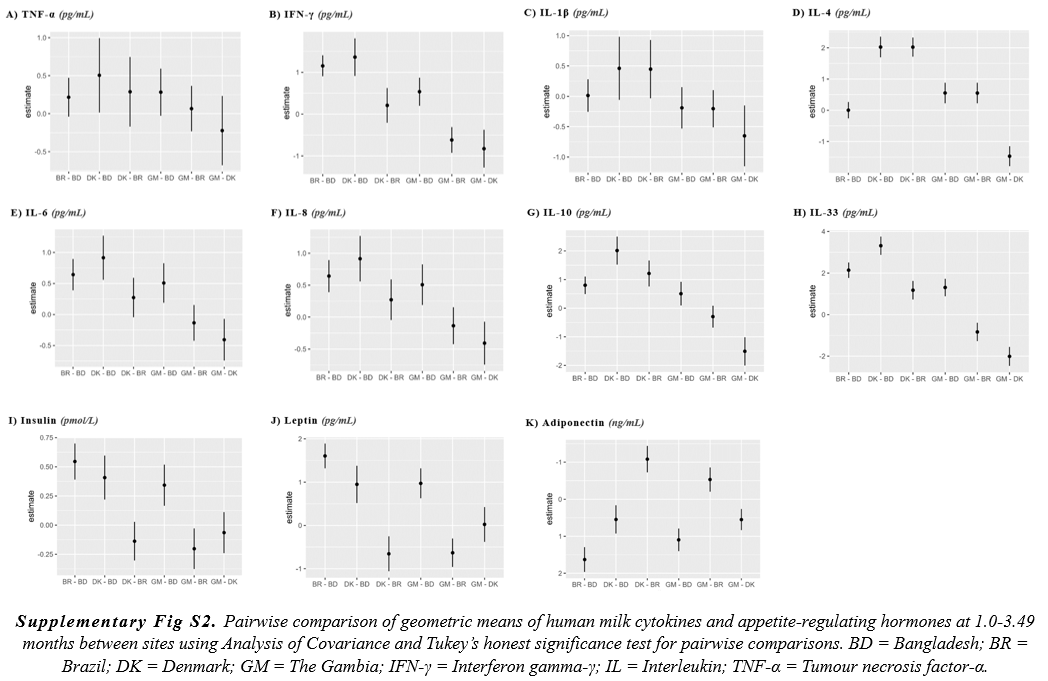

Supplement: S1 Table — (TIF) [file pone.0323204.s001.tif]

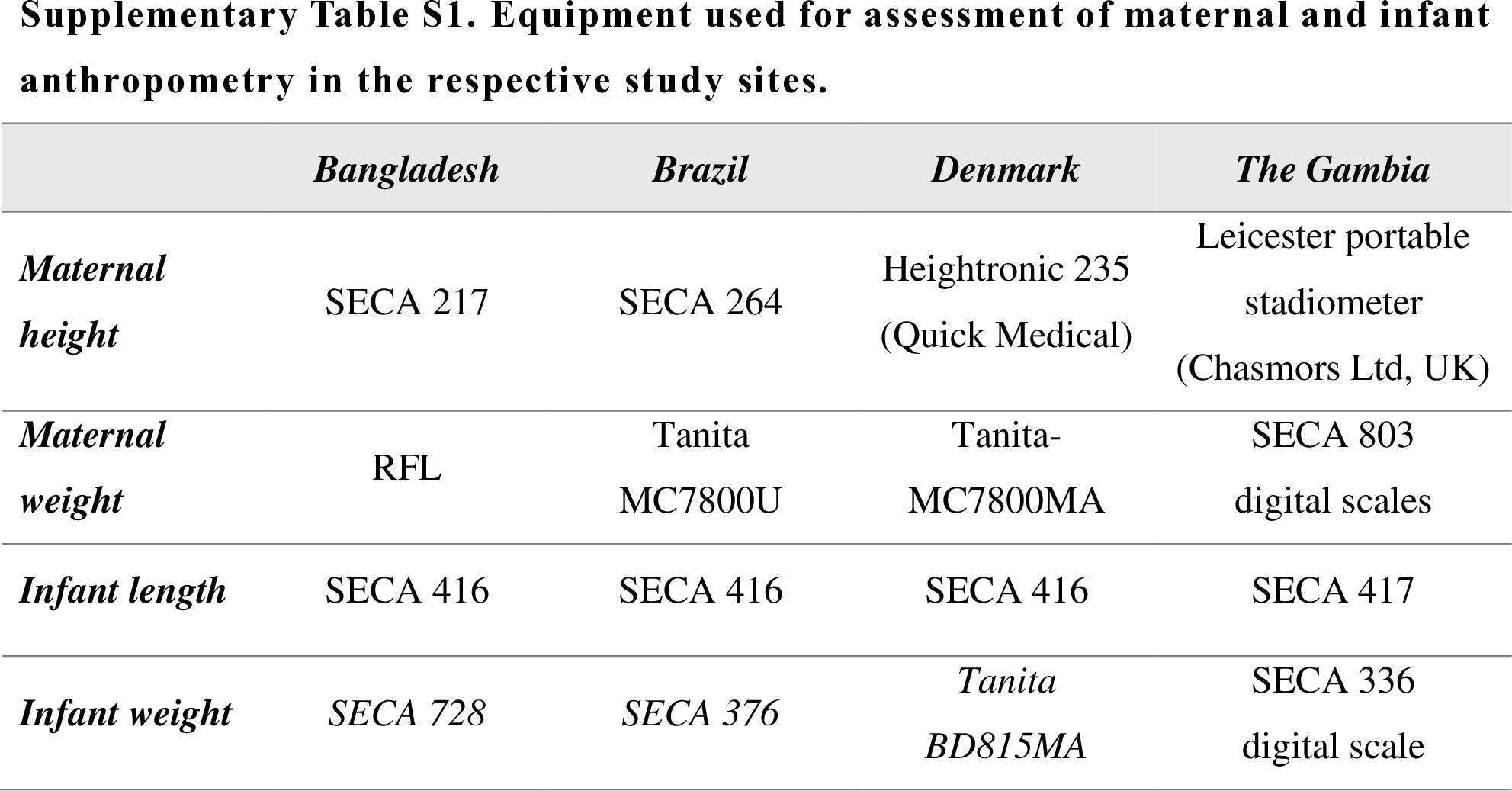

Supplement: S2 Fig — BD = Bangladesh; BR = Brazil; DK = Denmark; GM = The Gambia; IFN-γ = Interferon gamma-γ; IL = Interleukin; TNF-α = Tumour necrosis factor-α. (TIF) [file pone.0323204.s002.tif]

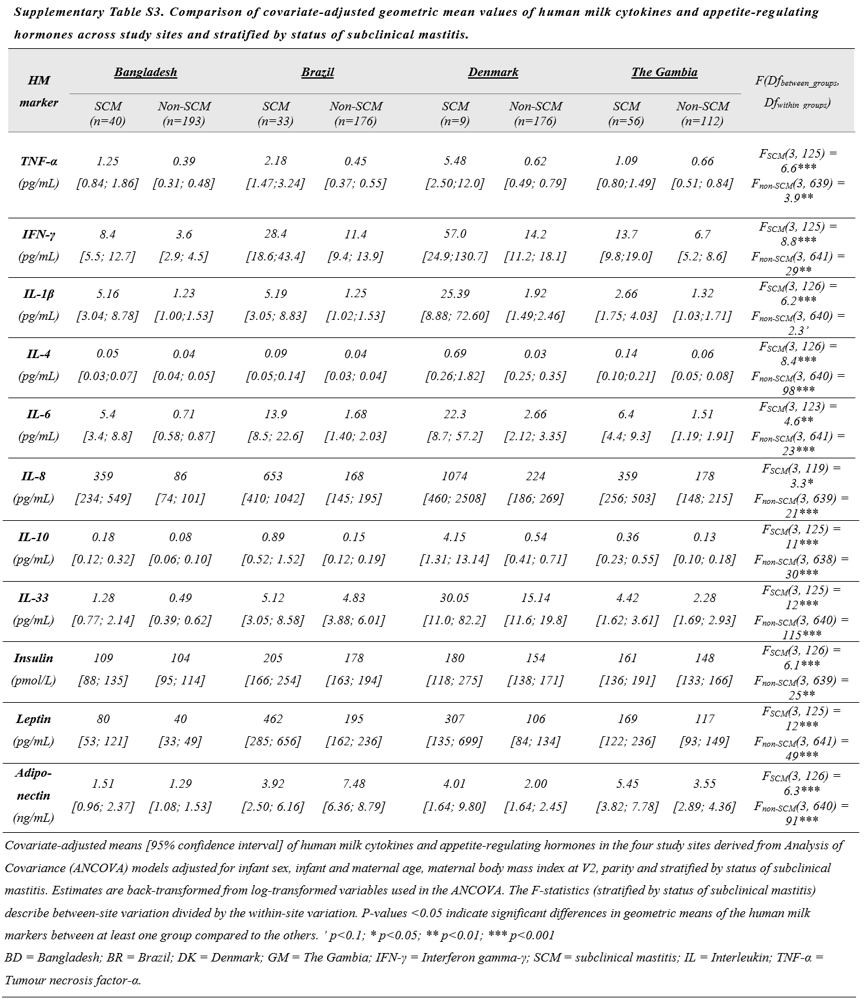

Supplement: S3 Table — (TIF) [file pone.0323204.s003.tif]
