# Supplementary material for: Cytokines and appetite-regulating hormones in human milk and associations with infant growth across four sites in a longitudinal cohort: The Mothers, Infants and Lactation Quality Study
Source: PLoS One. 2025 May 8;20(5):e0323204. doi: 10.1371/journal.pone.0323204 (PMC12061169; doi:10.1371/journal.pone.0323204)
Supplement: Attachment — Submitted filename: Comments .pdf [file pone.0323204.s004.pdf]

Major Comments

1. Study Design and Methodology

Clarity of Objectives: The objectives of the study are clearly stated, focusing on cytokine and appetite-regulating hormone concentrations in human milk and their association with infant growth. However, further elaboration on how these objectives align with existing literature would strengthen the introduction.

Sample Size Justification: While the manuscript mentions a sample size of 825 mother-infant dyads, it lacks a detailed justification for this size in terms of statistical power. Including a power analysis would enhance the credibility of the findings.

2. Data Collection and Analysis

Cytokine Measurement Techniques: The methods used for measuring cytokines and appetite-regulating hormones should be described in greater detail, including any standardization processes or validation methods that were employed.

Statistical Analysis: The use of analysis of covariance (ANCOVA) is appropriate; however, the manuscript should specify which covariates were included in the model. Additionally, it would be beneficial to discuss how potential confounding factors were controlled.

3. Results Interpretation

Geometric Means Reporting: The results section reports geometric means for cytokines and hormones, but it could benefit from including confidence intervals or standard deviations to provide a clearer picture of variability.

Contextualization of Findings: The differences observed among study sites are significant; however, the discussion should better contextualize these findings within existing research on environmental factors affecting human milk composition.

4. Infant Growth Associations

Z-score Calculations: The method for calculating Z-scores for infant growth should be explicitly detailed to ensure reproducibility. It would also be helpful to clarify how these scores were interpreted in relation to growth standards.

Causal Inferences: While associations between HM markers and infant growth are noted, caution should be exercised in making causal inferences without longitudinal data supporting these claims.

5. Ethical Considerations

Ethics Statement Clarity: The ethics statement is comprehensive but could benefit from a more concise summary of ethical approvals obtained across different sites to enhance readability.

6. Funding and Competing Interests

Transparency in Funding Sources: The funding disclosure is provided; however, it should explicitly state whether funders had any role in study design or data interpretation to maintain transparency.

7. Conclusion Strengthening

Implications of Findings: The conclusion summarizes the results effectively but could further emphasize the implications for public health or future research directions based on the findings regarding human milk composition and infant growth.
