# Supplementary material for: Cytokines and appetite-regulating hormones in human milk and associations with infant growth across four sites in a longitudinal cohort: The Mothers, Infants and Lactation Quality Study
Source: PLoS One. 2025 May 8;20(5):e0323204. doi: 10.1371/journal.pone.0323204 (PMC12061169; doi:10.1371/journal.pone.0323204)
Supplement: Attachment — Submitted filename: Response to Reviewers.docx [file pone.0323204.s006.docx]

**Rebuttal letter**

Thank you very much for your valuable comments, which improved our manuscript. We have answered your comments below and addressed them appropriately in the revised manuscript. We believe the manuscript has been improved and hope it is satisfying for the reviewers.

**Major Comments**

**1. Study Design and Methodology**

**Clarity of Objectives:** The objectives of the study are clearly stated, focusing on cytokine

and appetite-regulating hormone concentrations in human milk and their association with

infant growth. However, further elaboration on how these objectives align with existing

literature would strengthen the introduction.

We agree, and we have elaborated on this in line 96-99.

**Sample Size Justification:** While the manuscript mentions a sample size of 825 motherinfant

dyads, it lacks a detailed justification for this size in terms of statistical power.

Including a power analysis would enhance the credibility of the findings.

We understand this comment, however, we believe that a post hoc power calculation is not appropriate as this does not provide further information than the currently provided confidence intervals (e.g., Table 3 and Table 5) (for further information: <https://www.nature.com/articles/s41430-022-01169-4>). However, we acknowledge this issue can be enhanced and have elaborated on the aspects by also including aspects the width of the confidence intervals (line 444-447). We do hope this is sufficient.

**2. Data Collection and Analysis**

**Cytokine Measurement Techniques:** The methods used for measuring cytokines and

appetite-regulating hormones should be described in greater detail, including any

standardization processes or validation methods that were employed.

We have added a description of the use of internal standards and duplicate measurements in the Method section (line 173-177).

**Statistical Analysis:** The use of analysis of covariance (ANCOVA) is appropriate; however,

the manuscript should specify which covariates were included in the model. Additionally, it

would be beneficial to discuss how potential confounding factors were controlled.

We agree, and this is already described in line 222-223 (in the revised manuscript) (line 235-236 for linear regression models) as well as in the table text when appropriate. We hope this is sufficient.

**3. Results Interpretation**

**Geometric Means Reporting:** The results section reports geometric means for cytokines and

hormones, but it could benefit from including confidence intervals or standard deviations to

provide a clearer picture of variability.

Confidence intervals are provided in Table 3 presenting covariate-adjusted geometric means of cytokines and hormones from the ANCOVA models. We hope this is sufficient.

**Contextualization of Findings:** The differences observed among study sites are significant;

however, the discussion should better contextualize these findings within existing research on

environmental factors affecting human milk composition.

We agree and have elaborated on this matter in the discussion (line 383-392).

**4. Infant Growth Associations**

Z-score Calculations: The method for calculating Z-scores for infant growth should be

explicitly detailed to ensure reproducibility. It would also be helpful to clarify how these

scores were interpreted in relation to growth standards.

The Z-scores were calculated by using the WHO Anthro software, which we have described further and referenced in line 201-205.

**Causal Inferences:** While associations between HM markers and infant growth are noted,

caution should be exercised in making causal inferences without longitudinal data supporting

these claims.

We fully agree and have emphasized this in line 438-439.

**5. Ethical Considerations**

**Ethics Statement Clarity:** The ethics statement is comprehensive but could benefit from a

more concise summary of ethical approvals obtained across different sites to enhance

readability.

I am not sure about this comment. A ‘summary’ of the ethical statement is provided in line 107-111 in the manuscript, which we believe is sufficient. The ethics statement in the online submission is more comprehensive and include detailed information on the ethical documents from each site. Please let us know if further (or less) information is needed.

**6. Funding and Competing Interests**

**Transparency in Funding Sources:** The funding disclosure is provided; however, it should

explicitly state whether funders had any role in study design or data interpretation to maintain

transparency.

This has been added to the Funding Source (line 726-727)

**7. Conclusion Strengthening**

**Implications of Findings:** The conclusion summarizes the results effectively but could

further emphasize the implications for public health or future research directions based on the

findings regarding human milk composition and infant growth.

We agree and have added this aspect to the conclusion (line 501-506).
